# Image-Based High-Throughput Phenotyping in Horticultural Crops

**DOI:** 10.3390/plants12102061

**Published:** 2023-05-22

**Authors:** Alebel Mekuriaw Abebe, Younguk Kim, Jaeyoung Kim, Song Lim Kim, Jeongho Baek

**Affiliations:** Department of Agricultural Biotechnology, National Institute of Agricultural Science, Rural Development Administration, Jeonju 54874, Republic of Korea

**Keywords:** phenotyping, image analysis, phenomics, sensor, horticultural crop

## Abstract

Plant phenotyping is the primary task of any plant breeding program, and accurate measurement of plant traits is essential to select genotypes with better quality, high yield, and climate resilience. The majority of currently used phenotyping techniques are destructive and time-consuming. Recently, the development of various sensors and imaging platforms for rapid and efficient quantitative measurement of plant traits has become the mainstream approach in plant phenotyping studies. Here, we reviewed the trends of image-based high-throughput phenotyping methods applied to horticultural crops. High-throughput phenotyping is carried out using various types of imaging platforms developed for indoor or field conditions. We highlighted the applications of different imaging platforms in the horticulture sector with their advantages and limitations. Furthermore, the principles and applications of commonly used imaging techniques, visible light (RGB) imaging, thermal imaging, chlorophyll fluorescence, hyperspectral imaging, and tomographic imaging for high-throughput plant phenotyping, are discussed. High-throughput phenotyping has been widely used for phenotyping various horticultural traits, which can be morphological, physiological, biochemical, yield, biotic, and abiotic stress responses. Moreover, the ability of high-throughput phenotyping with the help of various optical sensors will lead to the discovery of new phenotypic traits which need to be explored in the future. We summarized the applications of image analysis for the quantitative evaluation of various traits with several examples of horticultural crops in the literature. Finally, we summarized the current trend of high-throughput phenotyping in horticultural crops and highlighted future perspectives.

## 1. Introduction

The world population keeps increasing and is expected to reach ten billion by 2050, so as the demand for food and energy. This alarms the need to maximize the yield and quality of food crops as well as reduce postharvest losses. Breeding for high yield, better quality, and resistance to biotic (disease, pest, weed) and abiotic (drought, salt, heat, cold) stresses should be the priority to meet the projected food demand. Plant phenotyping is the core of any plant breeding program, and accurate measurement of plant traits is essential for the selection of the best genotypes. Phenotype is the result of the interactions between genotype and all the surrounding environmental conditions during the plant growth cycle, whereas phenotyping refers to the measurement of any aspect of plant traits, including growth, development, and physiology [1]. Plant phenomics is the high-throughput collection and analysis of multidimensional phenotypes of the whole plant through its life span [2,3]. The advancement of next-generation sequencing and marker technology has accelerated genomic study, allowing the mapping and identification of genes controlling complex traits [4]. However, phenomic information is not adequately available due to the effect of environmental factors and lack of accurate measurements limiting the phenotypic characterization of crop traits.

Conventional phenotyping has been the bottleneck for breeding for a long time as it is labor-intensive, time-consuming, and does not have high throughput [5]. The recent introduction of high-throughput phenotyping methods is accelerating plant phenotyping, enabling high-throughput measurement of several phenotypic data nondestructively and objectively [3,6]. Furthermore, high-throughput phenotyping with the help of optical sensors, computer vision, and robotics will bring new traits under consideration which were difficult to measure via the conventional method.

High-throughput phenotyping platforms can image hundreds of plants daily using various types of optical sensors, allowing the measurement of morphological, physiological, biochemical, and performance traits in a non-destructive way [7,8,9,10]. The principle of image-based phenotyping is based on the interaction of electromagnetic radiation and the plant surface, including absorption, reflection, emission, transmission, and fluorescence, which differ between normal and stressed plants or among genotypes [9]. These interactions will help to estimate various types of phenotypic traits of the plant with the help of optical sensors. Image-based high-throughput phenotyping aims to quantify numerous traits, which requires the use of various types of optical sensors. Some of the currently available sensors for plant phenotyping include visible light (red–green–blue), thermal, fluorescence, hyperspectral, multispectral, light detection and ranging (LiDAR), magnetic resonance (MRI) imaging, X-ray computed tomography (X-ray CT), and positron emission tomography (PET) [3,11,12]. The applications of various types of sensors may differ depending on imaging platforms, accessibility, cost, and the target trait under consideration. The different sensors can be used separately or in combination for fast and accurate plant phenotyping, each of which comes with its own advantages and limitations.

High-throughput phenotyping is used in breeding, crop cultivation, and even postharvest, depending on the purpose of phenotyping. In plant breeding, phenotyping a large number of samples (population) aims to increase the selection intensity and accuracy and characterize various crop traits to select the best genotypes, while phenotyping in crop cultivation is used to monitor the occurrence of any plant stresses such as disease, pests, nutrient stress, weeds, or abiotic stress at early stages [1,8]. Real-time phenotypic data acquisition and analysis will help to make immediate management decisions for the crop. Hence, image-based phenotyping will play a pivotal role in the precision cultivation of horticultural crops. Horticultural crops are mostly utilized in the fresh state and are highly perishable due to their high water content, such as vegetables and fruits. The market value of horticultural products is highly dependent on external appearance (color, shape, size, texture) and internal (soluble solid content and firmness) quality attributes. The status of these quality attributes changes over time during maturity, ripening, and postharvest storage and should be routinely monitored [13,14]. Currently, external quality attributes are mostly evaluated using visual inspection in the horticulture chain, which is slow and subjective. On the other hand, internal quality attributes are quantified using destructive laboratory analysis or handheld/portable instruments, which are also limited in speed and sample size. Due to the highly perishable nature and the dynamics of quality attributes over time in horticultural products, image-based phenotyping will greatly improve the speed, volume, and accuracy of postharvest phenotyping.

**Figure 1:**
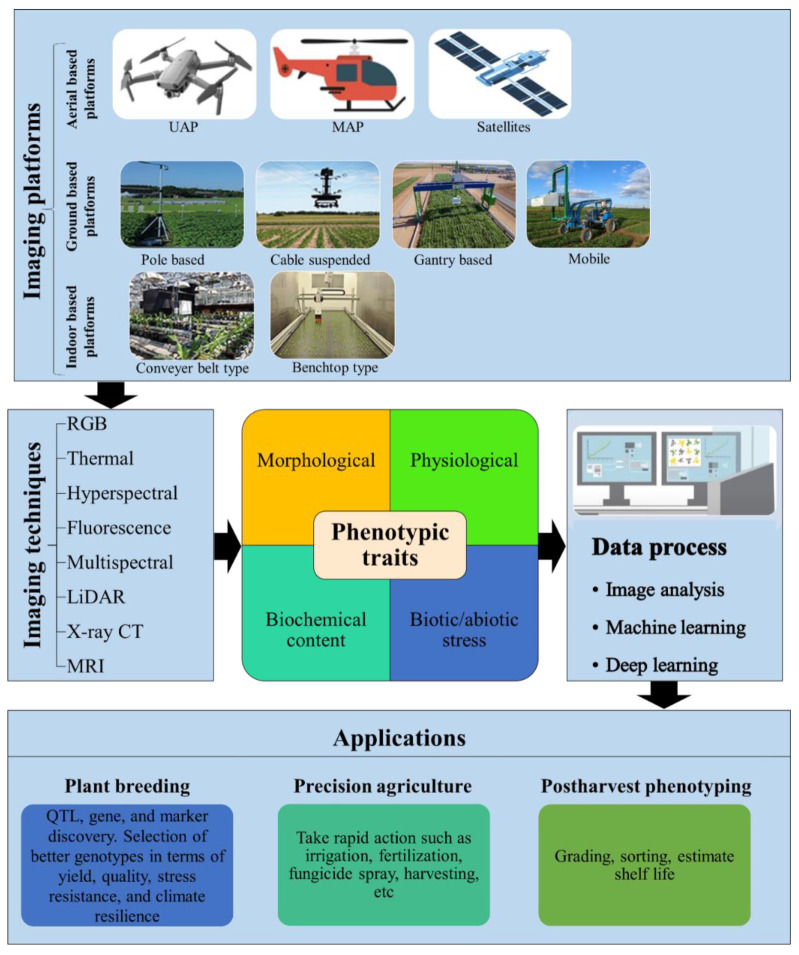
Schematic overview of image-based high-throughput phenotyping in horticultural crops. UAP, unmanned aerial platform; MAP, manned aerial platform; RGB, red–green–blue; LiDAR, light detection and ranging; X-ray CT, X-ray computed tomography; MRI, magnetic resonance imaging.

In this paper, we reviewed the applications of image-based phenotyping for the assessment of various traits in horticultural crops. Commonly used imaging platforms and sensors for high-throughput phenotyping are described. The application of these technologies for phenotyping various traits of horticultural crops is discussed with several examples in the literature. Finally, the current trends and future perspectives of high-throughput phenotyping in horticultural crops are highlighted. Using multiscale imaging platforms equipped with state-of-the-art imaging technologies will enable rapid and accurate quantitative measurement of diverse plant phenotypic traits to accelerate crop improvement, precision agriculture, and objective postharvest phenotyping (Figure 1).

## 2. High-Throughput Phenotyping Platforms

High-throughput phenotyping is carried out using various types of imaging platforms. The suitability of high-throughput phenotyping platforms may vary depending on the imaging environment, e.g., laboratory, growth chamber, greenhouse, or field. In most controlled environment conditions, imaging is carried out by moving the sensor towards the plant (sensor movement type) or the plant is transported to the fixed imaging setup using a conveyer belt or other transporting methods (plant movement type) for routine phenotypic measurement. For example, a greenhouse-based sensor-to-plant platform was used to measure static and dynamic traits such as geometric, structural, color, and textural phenotypes of lettuce [15]. Image-based phenotyping in controlled environment conditions has the advantage of high precision, high repeatability, continuous automated operation, and absence of interference from external environmental conditions. However, they are generally expensive and can monitor a very limited number of samples. The conveyer type and benchtop-type platforms are mostly used and have well-established systems for controlled environment conditions [6].

Imaging platforms for field-based conditions can be ground or aerial-based, targeting phenotyping of plant characteristics at individual plant or area levels. They are grouped into ground-based or aerial-based on the structures where the sensor is mounted. Ground-based imaging platforms such as pole/tower-based, mobile platform (vehicle), gantry-based, and cable suspended are flexible for deployment and have a good spatial resolution. However, they are subject to varying environmental conditions due to the slow speed of covering a large field. Aerial-based imaging platforms include unmanned aerial platforms, manned aerial platforms, and satellites. These imaging platforms can cover a wide area in a short period of time and are able to overcome varying environmental conditions. The disadvantage of the platforms is that they have a limited payload, and the spatial resolution of the image is affected by the speed and altitude of the aerial structure [6,9]. Unmanned aerial vehicle (UAV) platforms were used for the measurement of various traits in horticultural crops. For example, UAV-based remote sensing coupled with different machine learning approaches was used for disease detection and classification in potato, tomato, banana, pear, and apple [16,17,18,19,20,21,22], for tree detection in orchards such as banana and citrus [23,24,25], for aboveground biomass estimation in onion, potato, tomato, and strawberry [26,27,28,29], and other traits of fruits and vegetables [23,30,31].

## 3. Commonly Used Imaging Techniques for High-Throughput Plant Phenotyping

### 3.1. Visible Light Imaging

Visible light sensors detect light in a wavelength spectrum of 400–700 nm and provide reflected values of red, green, and blue (RGB). Visible light imaging is widely used for high-throughput phenotyping because of its accessibility, simplicity, and low cost [32]. High-resolution RGB images can be used to accurately measure plant biomass [28,33,34,35,36,37], root architecture [38,39], plant growth rate [40,41,42,43], germination rate [44], yield [45,46,47], disease detection and quantification [17,48,49,50], and abiotic stresses [51]. Their application in the field can be affected by minimal color variation between the leaf and the background and the influence of light for automatic image processing [32].

### 3.2. Thermal Imaging

Thermal infrared imaging allows the visualization and distribution of infrared radiation over a leaf or plant surface. A thermal camera converts infrared radiation (heat) emitted from the object into visible images showing the spatial distribution of surface temperature. The thermal sensor records the emitted light from the object in the thermal range of 3–5 μm or 7–14 μm with an image showing the temperature values per pixel. Thermal imaging can be used to detect the physiological status of the plant in response to biotic and abiotic stress, such as canopy or leaf temperature [52], transpiration and stomatal conductance [53], and plant water status [9,11]. Under water deficit conditions, plants close their stomata and reduce water loss through transpiration which is also highly linked with the soil moisture content. The reduction in transpirational cooling results in increased leaf temperature. Hence, thermal imaging can be used to manage water and irrigation in precision agriculture [54].

### 3.3. Hyperspectral Imaging

Hyperspectral imaging captures electromagnetic spectra (λ) and spatial (*x*, *y*) data at every pixel in an image to reconstruct the 3D data matrix called hypercube, containing thousands of images in the spectral range of 250–2500 nm encompassing UV, VIS, NIR, and SWIR [55]. It offers a large amount of information, allowing the extraction of a wide range of phenotypic traits, while the storage and analysis of the vast amount of hyperspectral data is challenging. Some of its applications include estimation of nutrient content, disease detection [16,56,57,58], fruit maturity and ripening [59,60], and other physiological and biochemical traits which are used to infer plant growth and development as well as yield [55].

### 3.4. Fluorescence Imaging

The light energy absorbed by chlorophyll can be used for photosynthesis, dissipated as heat, or re-emitted. Fluorescence is the light emitted when the plant absorbs radiation of shorter wavelengths, mainly via the chlorophyll complex, and is very small (<3%) compared to the total amount of radiation emitted to the object from the light sources. The amount of re-emitted light (fluorescence) is a good indicator of the plant’s ability to utilize the absorbed light and is used to estimate the overall plant health status [61]. Fluorescence imaging is used to estimate photosynthetic efficiency and other associated metabolic processes of the plant affected by biotic and abiotic stresses [62,63,64,65]. The fluorescence pattern of plants under stress conditions will show an altered pattern compared to no stressed plants. Sensors sensitive to fluorescence are used to capture fluorescence signals after illumination of the plant or tissue with visible light, infrared, or UV light. Maximum quantum efficiency (*F*_v_/*F*_m_), non-photochemical quenching (energy dissipated as heat from photosynthetic reaction center), the effective quantum yield of PSII (ΦPSII or *F*_q_′/*F*_m_′), and relative electron transport rate are some of the parameters derived from chlorophyll fluorescence which are used to assess the physiological status of the plant in relation to different stress conditions, where *F*_m_ is maximum fluorescence of a dark-adapted leaf and *F*_v_ is the difference between *F*_m_ and minimum fluorescence from dark-adapted leaf (*F*_0_) [10]. The problem with fluorescence imaging in the field condition is that it does not specify the cause of signal changes in the plant, e.g., light, temperature, or other environmental factors [11,61].

### 3.5. Tomographic Imaging

**Figure 2:**
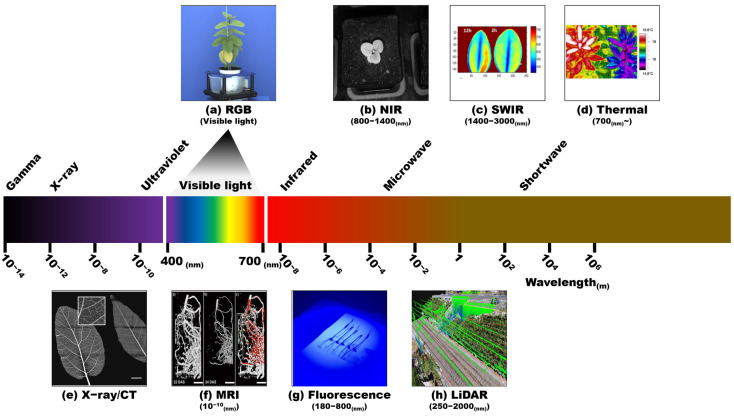
Examples of images from commonly used sensors in high-throughput phenotyping and their spectral range. (**a**) RGB; (**b**) NIR [71]; (**c**) SWIR [72]; (**d**) thermal (Qubit phenomics, Canada); (**e**) X-ray [73]; (**f**) MRI [66]; (**g**) fluorescence; (**h**) LiDAR and photogrammetry point cloud (Pix4D S.A., Prilly, Switzerland). RGB and fluorescence images were captured in our lab.

Other imaging techniques such as magnetic resonance imaging (MRI), X-ray computed tomography (X-ray CT), and positron emission tomography (PET) provide high-resolution 3D images of a single plant or plant parts [66]. MRI captures the 3D images of the internal structures of the sample enabling non-invasive quantification of both static and dynamic traits such as structural, biochemical, and temporal changes inside the plant. MRI can be used to monitor changes in growth and development (seed and bulb germination, seed development, fruit growth, and root growth), water dynamics within the plant, drought stress responses (drought stress, salt stress, cold stress, and heat stress), and the plant–pathogen interaction [67]. X-ray CT is used to visualize the 3D structures of internal and external features of the plant at the micro or macro level. When the X-ray beam passes through the sample, part of the beam is absorbed, and the remaining radiograph is recorded by the detector. Multiple 2D projections are recorded by moving the sample or the sensor, which are then used to reconstruct the 3D images [68]. For example, it has been used for the characterization of size and shape-related morphological traits of seed and fruit [69,70]. These imaging techniques are time-consuming and are not suitable when a large number of samples are under consideration. In addition, due to the larger size and heavy weight of the equipment it is not usable on aerial imaging platforms [55]. Examples of images from commonly used sensors in high-throughput phenotyping are shown in Figure 2.

Imaging technology is the primary component of high-throughput plant phenotyping as acquired phenotypic traits are determined by the type of sensor (imaging technique). Visible light (RGB) imaging and multi/hyperspectral imaging techniques are widely used to acquire morphological, physiological, biochemical, biotic, and abiotic stress-related traits. Fluorescence imaging and thermal imaging are used to capture the photosynthetic and surface temperature of the plant, respectively, which are physiological traits. LiDAR, X-ray CT, and MRI are mainly used to acquire morphological traits [3]. Different imaging techniques come with their specific advantages and disadvantages to capture certain plant traits. The summary of imaging techniques and measurable phenotypic traits with potential applications in high-throughput plant phenotyping is presented in Table 1. Among the variety of commercially available sensors for different imaging techniques, the choice depends on the cost, robustness, and other specifications of the sensor to capture the target trait [74]. Examples of different sensors used for high-throughput phenotyping of some horticultural crops are presented in Table 2. High-throughput phenotyping will benefit from the increasing capabilities and advancements of sensor technologies.

## 4. Applications of Image-Based High-Throughput Phenotyping in Horticultural Crops

### 4.1. Measurement of Morphological Traits

The morphological traits, including color, size, shape, and surface texture, determine the appearance of the produce and are used as quality parameters for visual inspection of horticultural crops. Although visual evaluation is a widely used nondestructive method for grading and sorting in horticultural crops, utilization of high-throughput phenotyping platforms is essential to obtain robust, faster, and objective results [14]. Nowadays, quantitative measurement of these traits (color, size, shape, and surface texture) using image analysis is increasingly used in different horticultural crops [13,84,85,86,87].

For example, grape berry color is a very important trait in grape breeding, which is qualitatively classified into six classes (green, yellow, rose, red, grey, dark red violet, or blue–black) according to the International Organization of Vine and Wine [88] or simply as noir (red, blue, or black) and non-noir (green or white). However, a qualitative assessment is very difficult to differentiate between noir and non-noir groups. Image-based phenotyping using different color spaces, RGB (red–green–blue), L*a*b (lightness, red–green, blue–yellow), and HSI (hue, saturation, intensity), allows for the easy discrimination of grape berry genotypes with different colors. RGB and HSI are able to separate within the noir and non-noir groups and enable the identification of minor QTLs controlling grape berry color, which were not identified previously using qualitative evaluation [89]. Quantitative measurement of strawberry fruit shape based on elliptic Fourier descriptors (EFDs) [90] and image analysis allowed the identification of two QTLs for shape via a genome-wide association study [84]. The fruit shape was highly correlated with the fruit length-to-width ratio.

The application of computer vision for shape quantification using images of sweet potatoes has shown that shape features, length-to-width ratio, curvature, cross-section roundness, and cross-sectional diameters, are highly predictive of shape classes. A neural network-based shape classifier was able to predict marketable (high market value) and non-marketable sweet potato classes with 84.59% accuracy [13]. In most food industries, quality is mainly assessed by experts based on subjective evaluation, which is very slow and inconsistent. The application of image-based phenotyping in the food industry is very important for fast, reliable, and objective evaluation. The browning of apple slices was quantified using color space, L*a*b, and textural features (entropy, contrast, and homogeneity) from the RGB images taken over time and showed that cv. Golden Delicious has less browning compared to Honey Crispy and Granny Smith [87].

In most horticultural crops, color, size, and texture are used as indicators of maturity and ripening. The maturity and ripening of plum and banana fruits were estimated based on these features using image analysis in which color was the dominant feature for the classification of maturity and ripening levels [91,92]. In general, color, size, shape, and texture are used to evaluate the external qualities of horticultural crops that greatly affect the market value of the produce. The applications of these quality attributes for the assessment of external qualities of horticultural crops based on hyperspectral imaging were previously reviewed [14].

### 4.2. Measurement of Physiological Traits

Physiological traits indicate the processes that occur within the plant, such as photosynthesis, transpiration, and canopy temperature. These traits determine how the plant is functioning under certain environmental conditions and are used to characterize the plant response to biotic and abiotic stresses, plant growth, and plant development [3]. Physiological traits can be quantified using various types of sensors, including RGB, ChlF, multispectral/hyperspectral, and thermal.

In horticultural crops, the physiological processes continue after harvesting (postharvest physiology). Postharvest physiology deals with the response of horticultural produce during postharvest storage and handling conditions along the processing or marketing chain. It determines the ripening, shelf life, and the quality of the produce. Due to their highly perishable nature, the quality and shelf life of horticultural crops is dependent on pre/postharvest handling and storage conditions [14]. Hence, high-throughput postharvest phenotyping is necessary for rapid, robust, and accurate measurement of ripening, shelf life, quality, food safety, and biochemical contents of the horticultural produce [86]. This will help to track the physiological status of the produce and make immediate decisions to avoid economic losses. For example, visual inspection and analytical methods such as spectroscopy and HPLC (high-performance liquid chromatography) analysis are widely used for fruit quality assessment, which is labor-intensive, destructive, time-consuming, and not robust. Therefore, using high-throughput methods which can accurately and efficiently measure fruit and vegetable quality attributes is essential. Chilling injury, one of the most common postharvest physiological disorder in horticultural products was detected using hyperspectral imaging and achieved more than 91% detection accuracy in apple, peach, and kiwi fruit [93,94,95,96].

### 4.3. Biochemical Component Analysis

Horticultural crops are rich sources of pigments such as anthocyanin, carotenoid, and chlorophyll, which serve as strong antioxidants and promote human health [31]. Quantification of these pigments has been mainly based on laboratory extraction, which is laborious and time-consuming. Handheld nondestructive devices such as chlorophyll meters and chroma meters were developed as an option to overcome destructive measurement, but they are still limited to be used in large-scale production or breeding programs. Hence, image-based phenotyping of pigment content is receiving increasing attention because it is nondestructive, robust, and fast. Anthocyanin, carotenoid, and chlorophyll content of red lettuce genotypes showed a high correlation with the vegetation indices calculated from images taken by remotely piloted aircraft [31]. The total carotenoid content in cassava root was estimated from the colorimetric indices extracted from the RGB images of root pulp. The total carotenoid content of cassava root showed a high correlation with color indices b* and chroma [97]. In addition, optical sensors can be used to nondestructively measure the amount of nutrients in the plant, such as nitrogen, phosphorus, and potassium, enabling accurate monitoring of plant growth and precise management of crop production [98].

### 4.4. Disease Detection and Quantification

Plant diseases are one of the challenges of crop production worldwide, causing significant yield loss every year. Early detection and accurate measurement of disease is a vital part of phytopathology and breeding [10]. Assessment of disease using conventional visual scores and laboratory-based analysis is time-consuming, laborious, and subjective. In recent years, rapid and high-throughput methods for the measurement of disease extent and severity have been widely used based on image analysis captured by various types of sensors [22,48,79,99,100]. High-throughput detection and quantification of disease is especially essential in horticultural crops which are prone to diverse pathogens during pre-harvest and post-harvest handling stages. Image analysis has been widely used for the detection and quantification of horticultural crop diseases such as apple scab [101,102], fire blight [20,21,56,103], powdery mildew [48,104,105,106], Fusarium wilt [22], bacterial blight [107], bacterial wilt [108], early blight, and late blight [19,99,109,110,111]. Image analysis can be used to closely monitor the plant health status as the disease infection can be detected at early stages before the development of typical symptoms. This enables us to take appropriate management measures to reduce the yield or quality loss.

### 4.5. Abiotic Stress Responses

Abiotic stresses are any kind of environmental conditions that affect normal plant growth and yield, such as drought, salinity, heat stress, and cold stress. Rapid and accurate phenotyping of plant responses to various abiotic stresses is essential to accelerate plant breeding programs dealing with the development of climate-resilient genotypes. Image-based high-throughput phenotyping is especially important when screening a large number of accessions. Various imaging techniques have been used to measure the response of horticultural plants to different abiotic stresses [82,112,113,114]. Hyperspectral images were used to detect heat stress tolerance in ginseng [115]. Cadmium stress in kale and basil was detected using high-throughput hyperspectral images. Among the vegetation indices analyzed, only the anthocyanin reflectance index was able to detect all levels of cadmium stress in both kale and basil. The anthocyanin reflectance index was significantly higher in cadmium-stressed plants than in the respective controls [116]. The applications of high-throughput phenotyping using image analysis to assess various traits in selected horticultural crops are summarized in Table 3.

## 5. Current Status and Future Perspectives

**Figure 3:**
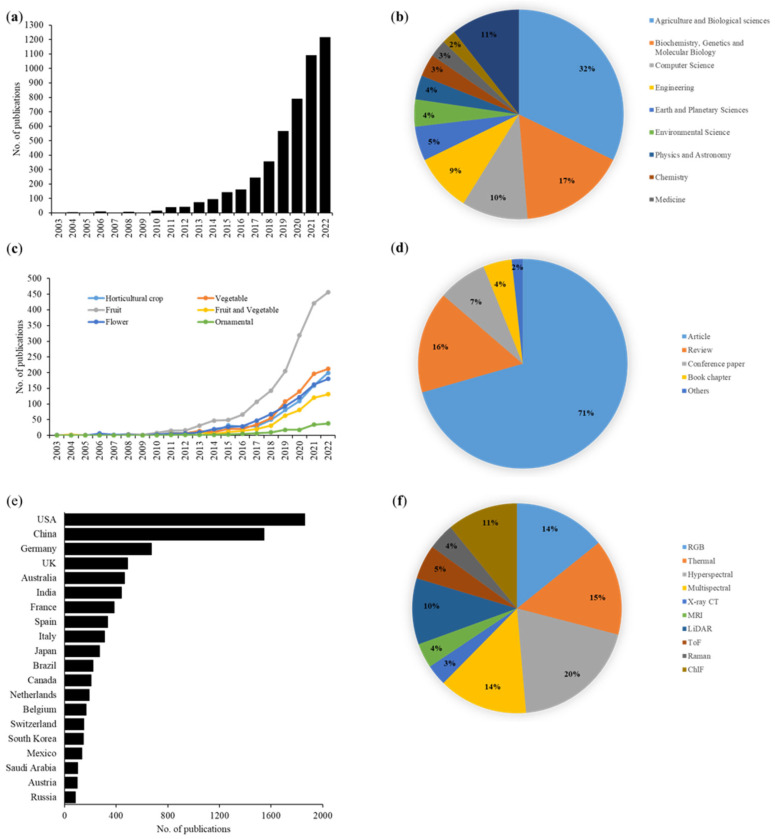
Statistics of image-based high-throughput phenotyping studies in horticultural crops during the past two decades. (**a**) The number of annual publications related to image-based phenotyping of horticultural crops. (**b**) Major areas of research using image-based high-throughput phenotyping. (**c**) Annual number of publications with different search keywords. (**d**) The type of publications related to image-based high-throughput phenotyping of horticultural crops. (**e**) Number of image-based high-throughput phenotyping studies by country (top 20). (**f**) Type of imaging techniques for high-throughput phenotyping of horticultural crops. Note: the data were obtained from the Scopus (https://www.scopus.com) database (Elsevier, The Netherlands) accessed on 21 February 2023. The publications were searched using keywords: horticultural crop, fruit, vegetable, ornamental, flower, and sensor types (RGB, thermal, hyperspectral, multispectral, X-ray CT, MRI, LiDAR, ToF, Raman, ChlF) within the search results of high-throughput phenotyping using image analysis.

The statistics of publications related to high-throughput phenotyping in horticultural crops for the last two decades were surveyed and summarized in Figure 3. The number of publications dealing with high-throughput phenotyping using image analysis is increasing every year and is mostly used in agriculture and biological sciences (Figure 3a,b). Among the search keywords, ‘fruit’ was the most mentioned word in these publications and showed an exponential increase in the last five years (Figure 3c), indicating the increasing interest of the researches to automate the measurement of various fruit traits during growth, maturity, ripening, and postharvest stages. Most of these documents are research articles (71%), followed by review papers (16%) (Figure 3d). Image-based phenotyping studies are actively conducted in many countries, with USA and China taking the lead with more number publications (Figure 3e). The applications of various types of sensors may depend on imaging platforms, accessibility, cost, and the target trait under consideration. Hyperspectral sensors are most popular for the phenotyping of horticultural crops, followed by thermal sensors (Figure 3f).

One of the upcoming challenges in phenomics is the handling of massive amounts of data generated from image-based phenotyping and the ability to extract important knowledge from big data [3]. Here, the application of computer science is inevitable when dealing with digital phenotyping. Specialized in handling multidimensional and multivariate data automatically, it is suitable for application to high-throughput phenotyping [190]. In machine learning (ML), humans are interpretable to the mathematical algorithm models that solve given problems such as classification, regression, and cluster. ML provides prompt results for classifying and identifying the plants or their phenotypes, predicting and estimating the yield or influences of external factors [191].

ML models start by training the dataset. Various algorithms/methods, such as support vector machine (SVM), decision tree, random forest, k-nearest neighbors (KNNs), logistics, regressions, clustering, dimensionality reduction, and artificial neural network (ANN), empower the training. All models require the accumulation of data for their accurate and efficient outputs [192]. Lack of sufficient data learning results in common errors in the output. This issue can be easily fulfilled by massive data processing from image-based high-throughput phenotyping.

However, when the amount of data to be processed is extremely large, deep learning will be more compatible than the other ML algorithms [193,194]. The machine learning applied ANNs is well known as ‘deep learning’. Deep learning has similar features but is slightly different from conventional ML types such as supervised learning, unsupervised learning, and reinforcement learning [195]. They differ depending on the absence or presence of human interventions in feature extraction after their training process. Deep learning requires no human intervention if the training data well annotates the targeted feature, while ML requires feature extraction before classification [196]. The most common algorithms used in deep learning are convolutional neural networks (CNN), long short-term memory networks (LSTM network), recurrent neural networks (RNN), multi-layer perceptron (MLP), and radial basis function networks (RBFN).

Despite their differences, both conventional MLs and deep learning provide powerful results. Therefore, appropriate selection based on the purpose and limitations would provide more advantages during the process. For instance, ML is generally used in the evaluation and prediction of stress, biomasses, and yields [16,28,171,173,186]. Deep learning is more proper in the detection, recognition, and counting of objects in large and complex datasets such as biotic/abiotic stresses and individual plants [197,198]. Recently, ML and DL methods have been increasingly used in high-throughput plant phenotyping of various traits (Table 4).

Available aerial high-throughput phenotyping platforms target the measurement of above-ground parts, while field-scale root phenotyping using images remains a bottleneck. Novel technologies enabling root phenotyping at the field level will be a breakthrough, especially for root and tuber crops, to capture root system architecture and to predict the yield of these underground parts at the field level. With the development of novel technologies with respect to sensing and data analysis methods, image-based phenotyping can discover new traits which were difficult to measure or detect using conventional phenotyping [3]. The newly discovered traits, in combination with the available omics data, need to be explored for the new frontier of crop improvement. The storage and sharing of the large amount of phenomic data obtained from image-based phenotyping are still challenging and need to be resolved. The data should be standardized and easily accessible among research communities, academia, industry, and farmers. Minimizing the cost of sensors and phenotyping platforms, along with the automation of big data analysis methods, will greatly increase the significance of phenomics in crop improvement to meet the projected global food demand.

## 6. Conclusions

Image-based phenotyping methods have become an integral part of plant breeding, cultivation, and quality assessment of economically important crops. This review highlights the progress and applications of image-based phenotyping as applied to horticultural crops. We explored commonly used imaging techniques: RGB, thermal, hyperspectral, fluorescence, and tomographic imaging with their advantages and drawbacks in relation to high-throughput phenotyping of horticultural crop traits. They are used to measure morphological, physiological, biochemical, disease and pest, and abiotic stresses.

In addition to accelerating the breeding cycle by enabling rapid and accurate measurement of phenotypic traits in a large population, image-based phenotyping will help to monitor the plant condition and make immediate decisions such as pesticide spray, fertilization, or harvesting, which will greatly improve yield and the quality of the produce. Moreover, the physiological processes of horticultural crops continue even after harvest, and their quality is highly dependent on postharvest storage and handling conditions. Hence, image-based phenotyping is especially important for postharvest phenotyping of horticultural crops. This will allow real-time monitoring of internal and external qualities of the horticultural product and will continue to play a significant role along the horticultural chain. Machine learning and deep learning technologies should be well integrated into image-based phenotyping to mine knowledge from the massive amount of data generated.

## Figures and Tables

**Table 1:** Summary of most common imaging techniques used in high-throughput plant phenotyping [11,75].

Imaging Technique ^a^	Phenotypic Traits	Advantages	Limitations	Potential Applications
Visible light imaging	Shape, color, size, biomass, pigment content, disease and pest, stress responses, nutrient stress, vegetation indices	Cheap, easy operation and maintenance, provide color information, high resolution, fast data acquisition	Limited to three spectral bands (RGB), affected by light, only provide relative measurement	Growth monitoring, plant stress detection, fruit maturity and ripening estimation, grading and sorting, quality evaluation, yield prediction, 3D modeling, crop management, robotic harvesting
Thermal imaging	Leaf greenness, leaf color, leaf chlorophyll content, leaf/canopy temperature, disease and pest, phenology, photosynthetic status	Wide measurement range, background interference can be removed	Require sensor calibration and atmospheric correction, difficulty of through time comparison due to changes in ambient condition affecting canopy temperature, need reference for comparison, difficult to separate soil and plant temperature in sparse canopies (limiting the automation of image processing)	Plant stress detection, irrigation scheduling
Hyperspectral imaging	Leaf/canopy water status, canopy coverage and volume, leaf greenness, disease and pest, photosynthetic rate, nutrient stress, metabolites	High spectral resolution, background interference can be removed	Expensive, low spatial resolution, too large image data challenging for storage and analysis, affected by ambient light condition	Growth monitoring, biotic and abiotic stress detection, fruit maturity and ripening estimation, quality evaluation, biomass estimation, metabolite prediction
Fluorescence imaging	Chlorophyll content, canopy coverage, disease and pest, photosynthetic status	Sensitive to fluorescence and water stress	Limited in field application, difficult to measure at the canopy scale due to the small signal-to-noise ratio	Growth monitoring, early detection of biotic and abiotic stress
Multispectral imaging	Canopy coverage and volume, chlorophyll content, leaf greenness, plant diseases and pests, photosynthetic status, water content	Easy in image processing; mature technology	Limited to several spectral bands; spectral data should be frequently calibrated using referenced objects; effects of camera geometrics, illumination condition, and sun angle on the data signal	Growth monitoring, biotic and abiotic stress detection
LiDAR	Plant height, canopy volume, shoot biomass	Provide three-dimensional shape	Expensive, sensitive to the small difference in path length; specific illumination required for some laser scanning instruments, data processing is time-consuming	Growth monitoring, structure capture
3D laser scanner	Geometrical plant traits such as shape, length, height, canopy structure and volume	Long measurement distance; high precision; good penetration	Expensive, affected by external factors such as wind and fog	Growth monitoring, organ morphogenesis
MRI	Internal structures, metabolites, development of root systems, water presence	Available for screening 3D structural information	Expensive, low throughput, slow data acquisition	Acquire 3D structures of the whole plant or plant parts
X-ray CT	Size and shape	Large penetration depth, scalable field of view, minimal sample preparation	Expensive, low throughput	Growth monitoring, seed and fruit development, organ morphogenesis, 3D visualization of plant organs and tissues

^a^ Imaging technique: LiDAR—light detection and ranging; MRI—magnetic resonance imaging; X-ray CT—X-ray computed tomography.

**Table 2:** Examples of sensors used for different imaging techniques in high-throughput plant phenotyping.

Imaging Technique	Sensor (Manufacturer)	Resolution	Crop	Reference
Visible light imaging	DJI Phantom 4 Pro (DJI Technology Co., Shenzhen, China)	3000 × 4000 px	Strawberry	[76]
	Sony Cyber-shot DSC-H3 camera (Sony Corporation, Tokyo, Japan)	3264 × 2448 px	Tomato	[38]
	Fujifilm X20 (Fujifilm Corporation, Tokyo, Japan)	3000 × 4000 px	Apple	[77]
Thermal imaging	3DR Solo quadcopter (3D Robotics, Berkeley, CA, USA)	1280 × 960 px	Banana	[23]
	Vario CAM hr inspect 575 (Jenoptic, Jena, Germany)	768 × 576 px	Mango	[78]
Hyperspectral imaging	Pika L 2.4 (Resonon Inc., Bozeman, MT, USA)	unknown	Tomato	[58]
	HySpex VNIR 1800, HySpex SWIR 384 (Norsk Elektro Optikk A/S, Skedsmokorset, Norway)	unknown	Grape	[79]
Fluorescence imaging	PlantScreen^TM^ Transect XZ system (Photon Systems Instruments, Drásov, Czech Republic)	1360 × 1024 px	Lettuce	[64]
Multispectral	Parrot Sequoia camera (Parrot Drone SAS, Paris, France)	1280 × 960 px	Banana	[80]
	RedEdge-M, (MicaSense, Seattle, WA, USA)	1280 × 960 px	Citrus	[81]
LiDAR	PlantEye F400 (Phenospex, Heerlen, The Netherlands)	unknown	Potato	[82]
3D laser scanner	FARO Focus 3D 120 terrestrial laser scanner (Faro Technologies Inc., Lake Mary, FL, USA)	1/5	Cassava	[83]
X-ray CT	X-ray imaging system (Xeye-5100F, SEC, Suwon, Republic of Korea)	2304 × 1300 px	Watermelon	[70]

**Table 3:** Applications of image-based high-throughput phenotyping in horticultural crops.

Crop	Trait	Sensor ^a^	Environment	Reference
Apple	Seed morphology	RGB	Laboratory	[117]
	Plant growth	RGB	Field	[40,118,119]
	Fruit detection and counting	RGB	Field	[77,120,121]
	Yield prediction	MS, RGB	Field	[45,122]
	Fruit ripening	Aerial video	Field	[123]
	Winter dormancy	ChlF	Field	[124]
	Low oxygen stress	ChlF	Laboratory	[125]
	Apple scab	Thermal, MS	Controlled	[101,102]
	Fire blight	MS, HS	Field	[21,56,103]
	Powdery mildew	RGB, MS	Field	[48]
	Drought stress	Thermal, MS	Field	[112]
Banana	Plant growth	Laser scanning	Field	[80,126]
	Plant counting	MS, laser scanning	Field	[23,126]
	Fruit maturity	RGB	Laboratory	[92]
	Yellow Sigatoka	RGB	Field	[17]
	Multiple diseases	RGB	Field	[49]
	Fusarium wilt	MS	Field	[22]
Cabbage	Seed morphology	RGB	Laboratory	[127]
	Plant growth	RGB	Field	[41]
	Shoot biomass	RGB	Field	[33]
	Heat stress	MS	Field	[41]
Carrot	Root morphology	RGB	Laboratory	[39,128,129]
Cassava	Root bulking rate	GPR	Field	[130]
	Root morphology	RGB	Field and Laboratory	[131,132]
	Shoot biomass	LiDAR	Field	[83]
	Root biomass	GPR	Field	[133]
	Carotenoid content	RGB	Laboratory	[97]
	Starch content	Thermal	Field	[134]
	Plant growth	RGB, MS	Controlled and field	[42,135]
	Bacterial blight	RGB	Laboratory	[107]
Citrus	Plant counting	RGB	Field	[24,81,136,137]
	Plant water status	Thermal	Controlled	[54]
	Citrus canker	HS	Field	[57]
	Huanglongbing (HLB)	GPR, MS, ChlF	Field	[62,138,139]
Grape	Bunch architecture	3D scanner	Field	[140,141,142]
	Berry counting	RGB	Field	[143,144]
	Berry maturity	RGB	Laboratory	[145]
	Yield prediction	RGB, HS	Field	[47,146,147,148]
	Grape yellows	HS	Field	[79]
	Grape leafroll	HS	Laboratory	[149]
	Powdery mildew	RGB	Laboratory	[104]
	Drought stress	RGB, Thermal	Field	[113]
Lettuce	Seed morphology	RGB	Laboratory	[150]
	Leaf semantic components	RGB	Laboratory	[151]
	Plant growth	RGB	Controlled	[43,152,153]
	Shoot biomass	ChlF	Controlled	[154]
	Anthocyanin content	RGB	Field	[31,155]
	Carotenoid content	RGB	Field	[31,156]
	Chlorophyll content	RGB	Field	[31]
	Drought stress	HS. ChlF	Controlled	[63,157,158]
	Salt stress	ChlF	Controlled	[64]
Mango	Fruit maturity	HS, LiDAR	Field	[159]
	Fruit ripening	HS	Field	[60]
	Fruit detection	RGB	Field	[30]
	Drought stress	Thermal	Field	[78]
Pear	Plant growth	RGB	Field	[160]
	Fire blight	HS	Field	[20]
	Russet	RGB	Laboratory	[50]
Pepper	Seed quality	X-ray CT	Laboratory	[161]
	Leaf area	RGB	Controlled	[162]
Potato	Crop emergence	RGB	Field	[44]
	Plant growth	RGB	Controlled	[163]
	Tuber growth and development	X-ray CT	Controlled	[164]
	Tuber skin color	RGB	Laboratory	[165]
	Tuber size	RGB	Laboratory	[166]
	Shoot biomass	RGB, HS	Field	[35,167,168]
	Nitrogen content	RGB	Field	[169]
	Tuber moisture content	HS	Laboratory	[170]
	Stomatal conductance	Thermal	Field	[53]
	Yield prediction	RGB, Thermal, HS	Field	[46,171]
	Early blight	HS	Field	[172]
	Late blight	RGB, MS	Field	[19,99,173,174]
	Bacterial soft rot	RGB	Laboratory	[51]
	Verticillium wilt	MS	Field	[18]
	Drought stress	LiDAR	Controlled	[82]
Strawberry	Plant growth	LiDAR	Field	[175]
	Fruit morphology	RGB	Laboratory	[84,176,177,178]
	Shoot biomass	RGB, Thermal	Field	[28,179,180]
	Yield prediction	RGB	Field	[76]
	Leaf gray mold	HS	Laboratory	[100]
	Verticillium wilt	RGB, MS	Field	[181]
	Heat and drought stress	HS	Controlled	[182]
Tomato	Root architecture	RGB	Controlled	[38]
	Shoot biomass	RGB	Controlled	[29,37]
	Fruit morphology	RGB	Laboratory	[183]
	Nitrogen, phosphorus, potassium content	MS	Controlled	[98]
	Chlorophyll content	RGB, MS	Controlled	[184]
	Yield prediction	RGB, MS	Field	[185,186]
	Bacterial wilt	ChlF	Controlled	[108,187]
	TYLC, early blight, bacterial spot	HS	Field	[16,58]
	Drought stress	RGB, Thermal, HS	Controlled, field	[188,189]
	Chilling injury (seedling)	ChlF	Controlled	[65]
	Salt stress	RGB, MS, Thermal	Field	[36]

**^a^** Sensor: RGB—red, green, blue; IR—infrared; HS—hyperspectral; MS—multispectral; NIR—near infrared; ChlF—chlorophyll fluorescence; LiDAR—light detection and ranging; GPR—ground penetrating radar.

**Table 4:** Examples of machine learning and deep learning applications in high-throughput plant phenotyping.

Algorithm Application	Algorithm Type	Imaging Technique	Plant Species	Phenotypic Trait	Reference
Classification	Faster R-CNN	RGB	Strawberry	Yield prediction	[76]
Classification	Convolution Network	X-ray CT	Watermelon	Seed quality	[70]
Classification	Linear Discriminance Model, Partially Least Square, Multi-Layer Perceptron, Radial-Basis Function Network	Hyperspectral	Grape	Grape yellows	[79]
Classification	YOLOv3	Multispectral	Hamlin citrus	Plant counting	[81]
Detection	3D Point clouds	Visible light	Apple	Fruit detection and counting	[77]
Detection	Convolutional Neural Network, Template Matching, Local Maximum Filter	Thermal	Banana	Plant counting	[23]
Identification	Neural Network Radial Basis Function, K-Nearest Neighbor	Hyperspectral	Tomato	bacterial spot	[58]
Identification	PCA	Fluorescence	Letucce	Salt stress	[64]
Identification	EVI2 Threshold	Multispectral	Banana	Plant growth	[80]
Identification	Logistic Regression	LiDAR	Potato	Drought stress	[82]

## Data Availability

Not applicable.
